# Somatoform disorder as a predictor of interstitial cystitis/bladder pain syndrome: Evidence from a nested case-control study and a retrospective cohort study

**DOI:** 10.1097/MD.0000000000006304

**Published:** 2017-05-05

**Authors:** I-Chun Chen, Ming-Huei Lee, Hsuan-Hung Lin, Shang-Liang Wu, Kun-Min Chang, Hsiu-Ying Lin

**Affiliations:** a Department of Psychiatry, Taichung Veterans General Hospital; b Department of Urology, Feng-Yuan Hospital, Ministry of Health and Welfare, Fengyuan District; c Central Taiwan University of Science and Technology, Beitun District, Taichung City, Taiwan; d Centre for Environment and Population Health, Griffith University; e Nathan Campus, Griffith University, Nathan QLD, Australia; f Department of Obstetrics and Gynecology; g Department of Anaesthesiology, Feng-Yuan Hospital, Ministry of Health and Welfare, Fengyuan Dist., Taichung City, Taiwan.

**Keywords:** comorbidity, interstitial cystitis/bladder pain syndrome, somatic symptoms

## Abstract

Interstitial cystitis/bladder pain syndrome (IC/BPS) has several well-known comorbid psychiatric manifestations, including insomnia, anxiety, and depression. We hypothesized that somatoform disorder, which is a psychosomatic disease, can be used as a sensitive psychiatric phenotype of IC/BPS. We investigated whether somatoform disorder increases the risk of IC/BPS.

A nested case-control study and a retrospective cohort study were followed up over a 12-year period (2002–2013) in the Taiwan Health Insurance Reimbursement Database. In the nested case-control study, 1612 patients with IC/BPS were matched in a 1:2 ratio to 3224 controls based on propensity scores. The odds ratio for somatoform disorder was calculated using conditional logistic regression analysis. In the retrospective cohort study, 1436 patients with somatoform disorder were matched in a 1:2 ratio to 2872 patients with nonsomatoform disorder based on propensity scores. Cox regression analysis was used to estimate the hazard ratio associated with the development of IC/BPS in patients with somatoform disorder, and the cumulative survival probability was tested using the Kaplan–Meier analysis.

We found that the odds ratio for somatoform disorder was 2.46 (95% confidence interval [CI], 1.05–5.76). Although the average time until IC/BPS development in the control subjects was 11.5 ± 1.3 years, this interval was shorter in patients with somatoform disorder (6.3 ± 3.6 years). The hazard ratio for developing IC/BPS was 2.50 (95% CI 1.23–5.58); the adjusted hazard ratio was 2.26 (95% CI 1.002–5.007). The patients and controls also differed significantly in their cumulative survival probability for IC/BPS (log rank *P* < .05).

Evidence from the nested case-control study and retrospective cohort study consistently indicated that somatoform disorder increases the risk for IC/BPS. Our study suggests that somatoform disorder can be used as a sensitive psychiatric phenotype to predict IC/BPS. Any past history of somatoform disorder should be documented while examining patients with IC/BPS.

## 1 Introduction

Somatoform disorder is characterized by somatic symptoms that predominantly involve pain accompanied by allodynia, which refers to the affective component of pain.^[1–4]^ The typical stimulus-evoking pain mechanism involves peripheral nociceptive sensory fibers, whereas the emotional and affective aspects of pain are processed by the reward/motivational circuits in the brain.^[4]^ Patients with somatoform disorder are also known to have comorbid panic and generalized anxiety disorder.^[5]^ These patients are evaluated based on the initial presentation of somatic symptoms and do not receive medical attention when treated by psychiatrists.^[6]^

Interstitial Cystitis/Bladder Pain Syndrome (IC/BPS) is a disease characterized by chronic pelvic pain, pressure, or discomfort perceived to be related to the urinary bladder accompanied by at least one other urinary symptom, such as a persistent urge to void or increased urinary frequency.^[7]^ There are numerous extra-pelvic somatic symptoms in patients with IC/BPS. These include fibromyalgia, irritable bowel syndrome, classical migraine, and depressive disorders.^[8]^ The most recent diagnostic guidelines for IC/BPS distinguish it from ulcer-type interstitial cystitis, type III interstitial cystitis, and Hunner's-type interstitial cystitis. These guidelines suggest the presence of 6 clinical phenotypes of IC/BPS: urinary, psychosocial, organ-specific, infection, neurological/systemic, and tenderness.^[9]^

Although previous studies have recommended that psychiatric manifestations be separately documented while assessing patients with IC/BPS,^[10,11]^ the comorbid psychiatric conditions described have been rather vague. These conditions include insomnia, anxiety, and depression.^[12]^ We, thus, aimed to identify a more sensitive phenotype of IC/BPS. Somatoform disorder is a more prominent feature of allodynia and alexithymia than anxiety disorder. We were, thus, driven to investigate the causal relationship between somatoform disorder and IC/BPS. We hypothesized that somatoform disorder is a sensitive psychiatric phenotype and aimed to investigate whether somatoform disorder increases the risk of IC/BPS.

## 2 Materials and methods

### 2.1 Study participants

**Figure 1:**
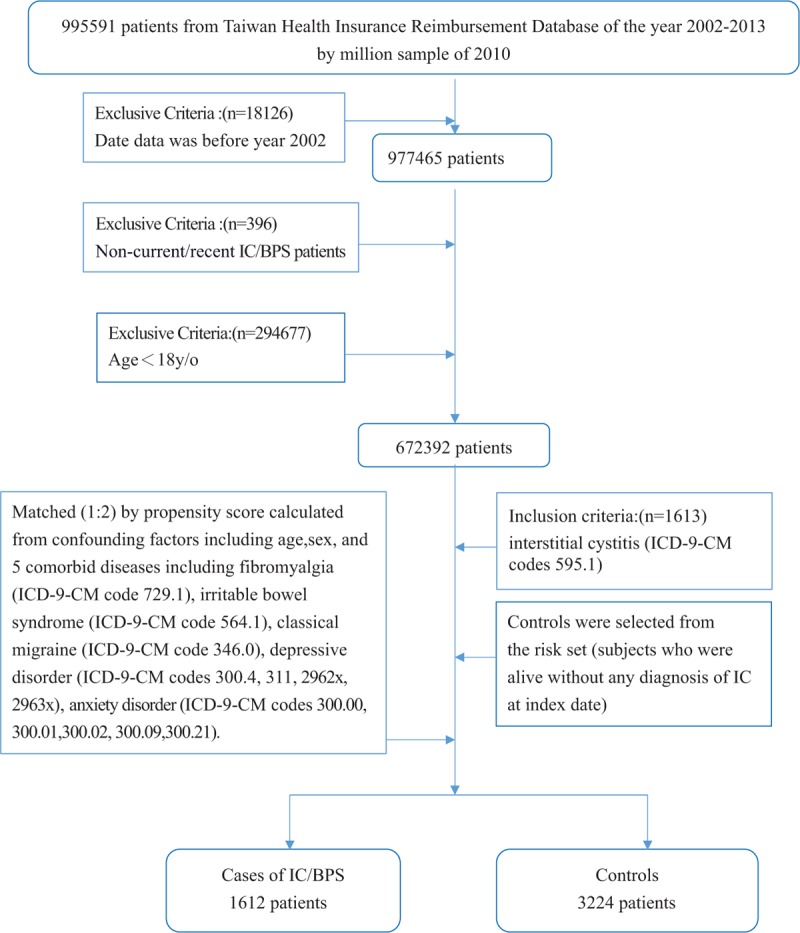
Flowchart of the retrospective cohort study.

**Figure 2:**
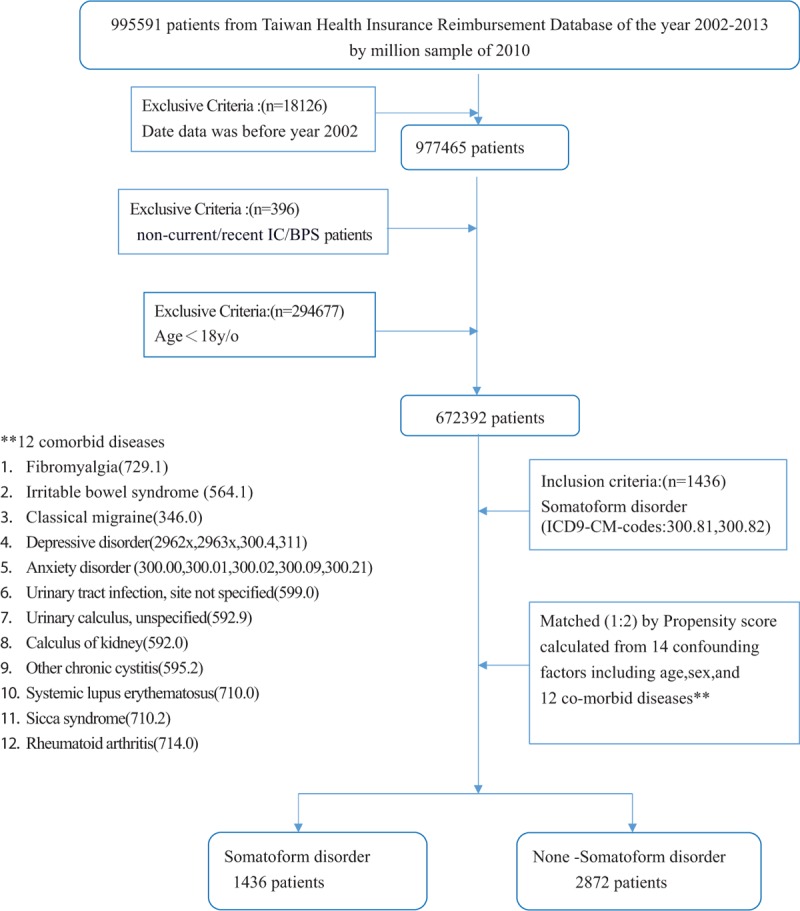
Flowchart of the nested case-control study.

The study population was selected from the Taiwan Health Insurance Reimbursement Database. The information used in this study was collected over a 12-year period (2002–2013). The dataset contained 1,000,000 patients who were randomly sampled from the Taiwanese population. Data from 995,591 patients were mined from the dataset. The exclusion criteria were (1) missing data (n = 18,126); (2) noncurrent/recent IC/BPS or non prevalent IC/BPS (n = 396), including IC/BPS diagnosed in 2002 or 2003, and IC/BPS diagnosed before somatoform disorder; and (3) age of younger than 18 years (n = 294,677). The study was conducted using information from the 672,392 patients that remained after patients meeting the above criteria were excluded (Figs. 1 and 2).

In the case-control study nested within these 672,392 subjects, the inclusion criterion was interstitial cystitis (International Classification of Diseases, Ninth Revision, Clinical Modification [ICD-9-CM] code 595.1) (n = 1613). Two controls for each case were selected from the *risk set* (subjects who were alive without any diagnosis of IC at the index date). Eligible controls were individually matched to cases based on age, sex, and co-variates influencing somatoform disorder, such as fibromyalgia (ICD-9-CM code 729.1), irritable bowel syndrome (ICD-9-CM code 564.1), classical migraine (ICD-9-CM code 346.0), depressive disorders (ICD-9-CM codes 300.4, 311, 2962x, 2963x), and anxiety disorders (ICD-9-CM codes 300.00, 300.01, 300.02, 300.09, 300.21). In this study, 1612 patients with IC/BPS were matched in a 1:2 ratio to 3224 controls. We then retrospectively studied these patients to determine whether they were diagnosed with somatoform disorder (ICD-9-CM codes 300.81 and 300.82) (Fig. 1).

In the retrospective cohort study, the inclusion criterion used was a confirmed diagnosis of somatoform disorder (ICD-9-CM codes 300.81 and 300.82; n = 1436; Fig. 2). Considering the previously reported antecedent nonbladder symptoms of IC/BPS, such as irritable bowel syndrome, fibromyalgia, chronic fatigue syndrome, panic disorder, depression, and migraine,^[13]^ the following 12 comorbid conditions served as co-independent variates in our study: fibromyalgia (ICD-9-CM code 729.1), irritable bowel syndrome (ICD-9-CM code 564.1), classical migraine (ICD-9-CM code 346.0), depressive disorders (ICD-9-CM codes 300.4, 311, 2962x, and 2963x), anxiety disorder (ICD-9-CM codes 300.00, 300.01, 300.02, 300.09, and 300.21), systemic lupus erythematosus (ICD-9-CM code 710.0), sicca syndrome (ICD-9-CM code 710.2), rheumatoid arthritis (ICD-9-CM code 714.0), urinary tract infection—site unspecified (ICD-9-CM code 599.0), urinary calculus—unspecified (ICD-9-CM code 592.9), calculus of kidney (ICD-9-CM code 592.0), and other chronic cystitis (ICD-9-CM code 595.2).

The propensity scores were calculated using 14 covariates, including sex, age, and the number of outpatient visits for each of the 12 comorbid diseases mentioned above (Fig. 1). Based on these criteria, 1436 patients with somatoform disorder were matched in a 1:2 ratio with 2872 patients with nonsomatoform disorders. The outcome exposure was the first date of outpatient visit for IC/BPS (ICD-9-CM code 595.1).

### 2.2 Statistical analyses

After matching the groups based on the propensity score, the 2 groups were examined to ensure that the sample was balanced. The baseline characteristics for each of the covariates were calculated using 1-way analyses of variance for continuous variables with normal distributions, and chi-square tests for categorical variables. In the nested case-control study, the odds ratio (OR) was calculated using conditional logistic regression analysis. In the retrospective cohort study, hazard ratios (HRs) were calculated using Cox regression analysis, whereas the cumulative survival probability was computed using Kaplan–Meier analysis. We calculated person-years for each study subject until IC/BPS was diagnosed, or until December 2013 for patients not diagnosed with IC/BPS. Results were considered significant when *P* was < .05. All statistical analyses were performed using the SAS software (SAS Institute Inc., Cary, NC).

## 3 Results

### 3.1 Nested case-control study

Data from 1612 patients with IC/BPS and 3224 controls were used in the study. The mean ages of the cases and the controls were 48.4 ± 16.4 and 48.9 ± 16.4 years, respectively (*P* > .05). The percentages of females in the case and control groups were 79.6% and 76.5%, respectively (*P* < .05). The total numbers of outpatient visits for fibromyalgia were 4.2 ± 8.8 in the cases and 5.3 ± 14.6 in the controls (*P* < .05). The total numbers of outpatient visits for depressive disorder were 6.8 ± 17.4 in the cases and 8.3 ± 23.1 in the controls (*P* < .05). There were no statistically significant differences in the total number of outpatient visits for irritable bowel syndrome, classical migraine, anxiety disorder, hypochondriasis, or conversion disorder (*P* > .05). The odds ratio for somatoform disorder was 2.46 (95% CI, 1.05–5.76), after adjusting for sex, fibromyalgia, and depressive disorder using a multivariate conditional logistic regression model.

### 3.2 Retrospective cohort study

Data from 1436 patients with somatoform disorder and 2872 patients with nonsomatoform disorder were used in the study. We found that there were significant differences between the 2 groups in age (*P* < .001) and sex (*P* < .05). However, the groups were evenly matched when considering the incidences of the 3 comorbid diseases with the highest numbers of outpatient visits during the 12-year follow-up period (anxiety disorder, depressive disorder, and fibromyalgia; Table 1). Given that the large sample size increased the probability of incorrect rejection of a true null hypothesis, we concluded that there were no systematic differences in the baseline characteristics of the 2 groups.

**Table 1:**
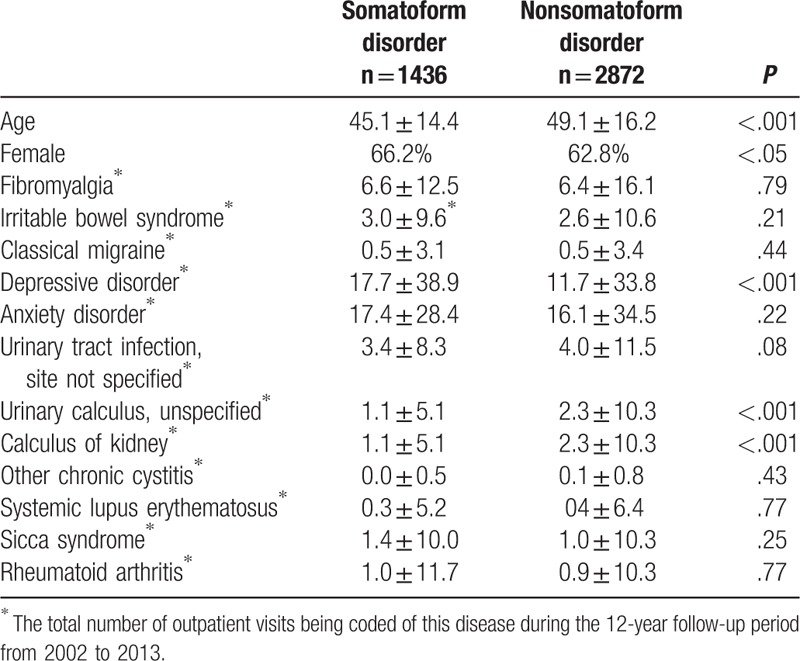
Baseline characteristics, including age, sex, and co-morbid diseases in 1436 patients with somatoform disorder and in 2872 patients with non-somatoform disorders matched in a 1:2 ratio by the propensity score.

**Figure 3:**
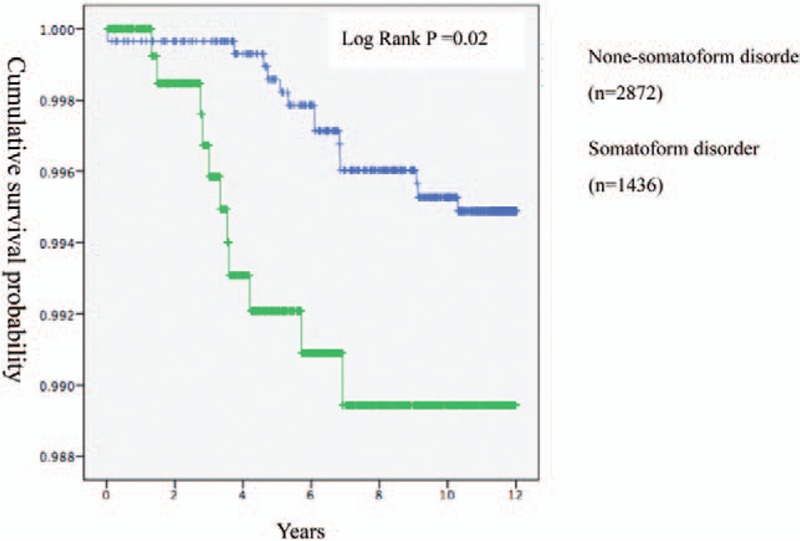
Kaplan–Meier analysis used to compute cumulative survival probabilities of IC/BPS in 1436 patients with somatoform disorder and 2872 patients with non-somatoform disorders matched in a 1:2 ratio by the propensity score. IC/BPS = Interstitial Cystitis/Bladder Pain Syndrome.

The average time after which patients with nonsomatoform disorder developed IC/BPS was 11.5 ± 1.3 years. This outcome was decreased to 6.3 ± 3.6 years in patients with somatoform disorder. When comparing patients with somatoform disorder to those with nonsomatoform disorders, the hazard ratio for developing IC/BPS was 2.50 (95% CI, 1.23–5.58) and the adjusted hazard ratio was 2.26 (95% CI, 1.002–5.007). In addition, the cumulative survival probability of IC/BPS in patients with somatoform disorder and that in patients with nonsomatoform disorders were significantly different (log rank *P* < .05, Fig. 3).

## 4 Discussion

To our knowledge, our study is the first nation-wide population-based longitudinal study to report an increased risk of IC/BPS in patients with somatoform disorder. Our rationale for designing the retrospective cohort study was that we could analyze the complete source materials from national health insurance reimbursement records for all cases and controls instead of only a relatively small number of cases and controls. We also designed a case-control study “nested” within the cohort. These 2 study designs had consistent findings and were reliable.

This study has numerous strengths. First, the nation-wide longitudinal dataset allowed the use of a large sample size and high statistical power. Second, since patients with somatoform disorder also have diagnoses of comorbid conditions, such as anxiety disorder and depressive disorder, we found collinearity between somatoform disorder and these comorbid diseases. The propensity score in our study accounts for each of these covariates and permits extensive control of collinearity among these variables.^[14]^ Third, our study included incident IC/BPS patients, allowing us to assess the temporal relationship between somatoform disorder and IC/BPS.

Naliboff et al^[15]^ have reported that patients with urologic chronic pelvic pain syndromes have more difficulty coping with illness, increased self-reports of cognitive deficits, and more widespread pain symptoms in the Multidisciplinary Approach to the Study of Chronic Pelvic Pain research network. Here, we investigated somatoform disorder, which also leads to difficulty in coping with illness and more widespread pain symptoms. Compared to the report by Naliboff et al, however, the longitudinal dataset used in our study allowed us to probe into the causal and temporal relationships between somatoform disorder and IC/BPS. We found that somatoform disorder is not just concurrent with IC/BPS, but also serves as one of the triggers for this condition. The link between somatoform disorder and IC/BPS is not simply due to an overlap of site-specific symptoms, which may lead to a misdiagnosis of early urinary organic pathology. Increasing evidence points to altered pain pathways in somatoform disorder and neuro-inflammation and altered nociceptive sensory inputs in IC/BPS.^[16,17]^ This association may be based on independent variables (e.g., genetic) related to proneness to depression rather than a pathophysiological bridge between standard nerve conduction abnormalities and peripheral complications. The medications used to treat somatoform disorder may explain the above findings. Treatment using tricyclic antidepressants, selective serotonin reuptake inhibitors, and serotonin norepinephrine reuptake inhibitors has been shown to be effective, although it leads to only incremental benefits.^[18,19]^ Recent studies offer insights into the interactions of the dopaminergic and serotonergic systems and increased risk of somatoform disorder.^[20]^ Antipsychotics augmentation, such as that achieved using dopamine partial agonists, has shown efficacy in the treatment of somatoform disorder.^[21]^

The groups had no significant differences in baseline characteristics for most covariates (9 out of 14). In a similar manner to the study by Allen-Brady et al,^[22]^ we identified IC/BPS and the associated baseline characteristics using the ICD-9 codes. The ICD-9 codes used to tag the comorbid diseases in the 2 studies are largely consistent and comprehensive. This bolsters the internal validity of the study. Therefore, we can conclude with confidence that the adjusted hazard ratio of 2.26 (95% CI, 1.002–5.007) computed in our analysis reflects a moderate-to-severe effect of somatoform disorder on the development of IC/BPS.

Chuang et al have reported that IC/BPS is a significant predictor for anxiety, depression, and insomnia. It is, therefore, reasonable to speculate that IC/BPS leads to psychological burden. Although it may appear controversial, we found that somatoform disorder markedly overlaps with anxiety, depression, and insomnia, and that these conditions are, in essence, the same. Although somatoform disorder predominantly involves pain, there are reports of patients with IC/BPS with changes in autonomic function^[23]^ and changes in provoked pain symptoms and mechanical pain thresholds over time.^[24]^ Therefore, the findings of our study are compatible with our hypothesis that somatoform disorder is a sensitive psychiatric phenotype of IC/BPS.

However, our study has some limitations. First, we only considered patients older than 18 years, which considerably reduced the external validity of our study. However, we excluded this population because the majority of these patients present with recurrent abdominal pain, noncardiac chest pain, and nonepileptic seizures in pediatric clinics, and are rarely diagnosed with somatoform disorder. Second, we created risk sets that were matched based on age, sex, and co-morbid diseases instead of using incidence density sampling (matching cases and controls based on person-time at risk). Third, the pre-existing health insurance reimbursement data were not necessarily acquired in a predetermined manner to assess risk factors for IC/BPS, although we accounted for comorbid diseases that may have influenced the development of IC/BPS. It might be important to know whether the subjects had psychosocial stressors or biological constitutions liable to IC/BPS. Finally, the groups were not similar in terms of sex and age in the retrospective cohort study.

In conclusion, our report suggests that somatoform disorder is a sensitive psychiatric phenotype that may be used to predict IC/BPS. Thus, past history of somatoform disorder should be documented when assessing patients with IC/BPS. In addition, patients and physicians should also investigate any unpleasant urinary bladder symptoms in patients with somatoform disorder, as they are currently not provided with specialized treatment by clinicians in any particular medical discipline.
